# False Impressions

**Published:** 1889-02-16

**Authors:** Caroline Fothergill

**Affiliations:** Author of "An Enthusiast," "A Voice in the Wilderness," etc.


### False Impressions.

By Caroline Fothergill, Author of "An Enthusiast," "A Voice in the Wilderness," etc.

(Continued from p. 305.)

Chapter XIX.—Visiting the Sick.

On the surface, a dinner party in private life seems a trifling thing enough, yet the one just chronicled was momentous, in that it led to complications of a very painful nature in the lives of four persons. It put the finishing touch to George's feeling for Beatrice, against which he had hitherto fought more or less. He had tried to go back to his old superstitions, to doubt the evidence of his own senses, to think of her as he had done in the beginning, before he knew anything about her. He had tried every means of extinguishing the glow which filled his heart whenever he thought of her, but in vain. Like most repressed fires, it only broke out more fiercely when it did get the chance, and at last on this evening which has just been described, when the guests had all gone, and he was alone in the silence and solitude of his study, he threw all his scruples and doubts to the winds, and resolved to yield wholly to his feeling for her. She was irresistible; it was useless to fight against her any longer; he was conquered. Then he drew a long breath of relief, and felt happier than he had done for many weeks, or even months.

Beatrice had gone home in a very similar frame of mind, outwardly calm, but inwardly tremulous with the feeling that George was stronger than she was, and that it would be a mere farce playing at indifference any longer. To herself she at last confessed the truth.

Mrs. Lester, too, had had all her suspicions and fears confirmed. She felt her pedestal tottering beneath her, and she resolved desperately to save it from falling, let the means she had to employ to that end be what they might.

Harry Fairfax had sought his lodgings with the knowledge that his future happiness lay in the little fragile hands of Edith Lester; and he, too, formed a bold resolve that if she was to be won he would win her. He knew her circumstances pretty accurately—that she was the penniless daughter of a poor London parson with a large family of growing, hungry boys, of whom the two eldest alone were even partially supporting themselves, and he was of opinion that if, after the tenderest and most respectful wooing that any man could give to any woman, she would consent to marry him, she would make the most charming clergyman's wife imaginable.

To Edith the evening had brought little pleasure. After the disagreement which she had witnessed between her aunt and George, she had made up her mind in private that she would devote the greater part of the evening to attentive observation of this highly interesting couple. Her intention had been frustrated by Harry Fairfax's attentions, which for the first time she had felt to be irksome. George, too, had not once addressed her during the evening, and although she was too modest to feel aggrieved, she did feel hurt and a little sad at the neglect; she made all possible excuses for him, but still deep down in her heart lurked the unspoken reproach, "he might have spoken to me once." Her greatest misfortune, however, was yet to come, and of it, happily or unhappily, she was still in ignorance. As at present her story is the most eventful, we will continue it.

A day or two passed by without anything worthy of note occurring, and then Edith met Harry, or rather, he crossed the street to speak to her. She was bound on a charitable mission for her aunt, whose deeds of mercy and kindness were usually carried out by someone else, in accordance with commands issued from her couch or easy chair. An old servant who had retired from active life, and was living in a little cottage on a comfortable pension provided by George and dispensed by his sister, who somehow managed to get credit for the provision of it as well, was suffering from a severe and prolonged attack of rheumatism. It was doubtful if she would ever leave her bed again, and Mrs. Lester, who liked to have the credit of doing things handsomely when, as in this case, it involved neither trouble nor expense, sent Edith to her every day with a little basket of dainties, suitable to her invalided condition, and instructions to read to her out of the Bible if she wished.

It was on the way to this old woman's cottage that Harry met her, as has been said, on the other side of the street; but so charming did she look in her fresh cotton dress and shady hat that he could not resist the temptation of crossing over to speak to her.

"Where are you going to?" he asked, when the first greeting had been exchanged. It had at once come into his mind that he might find a plausible excuse for turning back with her.

She told him her errand, adding, with a little sigh—

"It is so far and such a hot afternoon, and the basket is heavy, because I am taking her something which has to be kept in the dish it was made in."

Here was an opportunity not to be missed, and Harry liked it all the better because of the evident innocence with which it was given; it was quite clear that Edith had no idea of the opening she was giving him.

"If you will let me," he said at once, "I will go with you and carry the basket. I can see it is much too heavy for you."

"Oh, no, thank you," said Edith, blushing vividly; "there is no need to trouble you. I can carry it quite well, even if it is a little heavy."

"But you must not carry heavy weights, you are not strong enough; you might hurt yourself. Let me feel it," he went on, artfully contriving in this way to get the basket into his own possession. "It is much too heavy," he said at once, with decision; "suppose you dropped it and broke the dish. What would Mrs. Lester say?"

"She would be very angry," said Edith, with a simplicity and almost an awe, which rather amused her companion, even while he thought it perfectly charming.

"You would get into a regular scrape, and there would be no end of a row," he said confidently. "You would break the dish, you know. I am very glad I happened to be coming along It was a perfect Providence."

He turned, as he spoke, to walk at her side, but Edith looked very uncomfortable, and remained where she was.

"I am taking you all out of your way," she murmured, "and taking up your time, and there is no need. Aunt Frances would be very angry if she knew."

"Oh, no she wouldn't," said Harry, cheerfully; "I would explain it to her, and make her understand that one of her best dishes was in peril. Come along. Besides, I can talk to this sick old woman; that is proper parson's work—she can't object to that."

Edith said no more. Harry had begun to walk on in the direction of Mrs. Jones' cottage, for she had told him where it was, and as he had the basket she had perforce to follow him. In spite of the boyish manner she had begun to despise so much, he had made her do what he wanted, although she accompanied him half unwillingly. She had been lost in a dream about George, and did not like to be disturbed.

"Tell me more about this old woman," said Harry. "I do not know her, and if she is in our parish I ought to visit her regularly. She will feel she has been neglected if neither Atkinson nor I go to see her." (Mr. Atkinson was Harry's rector.)

"She is in the parish, I know," answered Edith, "because I remember Aunt Frances said she must mention her to Mr. Atkinson. But I don't think she has done. She forgets, and Mr. Atkinson does not call very often at our house; I suppose he has a great deal to do."

"A very great deal," said Harry with emphasis, for he did not wish to be deposed from the office of Mr. Atkinson's representative in Queen Street. "There will be no need to tell him now. I can see how she is, and tell him about her myself. If she seems to like me to-day, I can do the visiting for him. Atkinson has a great deal on hand just now."

"But it will be giving you extra work," ventured Edith.

"Oh, that does not matter," said Harry, generously. "I am young and strong, and never tired; Atkinson is getting an old man. I like to spare him as much as possible."

Edith did not seem as much impressed with this noble sentiment as she would have been a month or two earlier, and Harry went on:

"Do you come here every day?"

"Yes, just now, every day. She wants a good many things."

"And always at the same time?"

"Yes, it is the hottest part of the day; but Aunt Frances is having her afternoon sleep, and can spare me best. In the morning, or later than this, she wants me a great deal."

She saw nothing of the deep plot which her companion was laying, but answered his questions with a straightforwardness which increased Harry's admiration for her. She was also so accustomed to falling in with her aunt's wishes, that she did not see in what an unfavourable light her last words placed that lady. But Harry saw, and he muttered some words the reverse of complimentary to Mrs. Lester, and which would have shocked both her and Edith if they had heard them.

"I have not seen you since the dinner," he said aloud, "What a pleasant evening that was; I don't think I ever enjoyed myself more."

"Did you?" said Edith; "I thought it so dull. It was my first dinner party, and I do not mind if it was my last."

This was ungracious from Edith to Harry, but he remembered that she had been carrying a heavy basket in the hot sun, and asked:

"Why did you not enjoy it?"

By this time Edith had realised her rudeness, and she said in an apologetic tone:

"Oh, I did enjoy it, but I did not know dinner parties were so formal, or that there would be so many dishes I had never seen before. I felt nervous all the time, and afraid of doing something wrong."

"You were not likely to do anything wrong," said Harry chivalrously. "You always seem to do right by instinct; and if you had done wrong, it would have seemed better than anyone else's right."

Edith laughed a little, but said nothing; and Harry went on—

"That was a very beautiful lady who sat by Mr. Murray. I did not catch her name: who was she?"

"Miss Stanford," answered Edith, with a little reserve. "Did you think her so very beautiful?" she asked, after a pause.

"Yes, very!" said Harry, innocently. "I thought how well she and Mr. Murray looked together, and how well they seemed to get on. I heard other people say so, too."

Edith did not speak at once. These words gave her a stab; in spite of the heat, a little chill passed over her. Then she pulled herself together and said—

"I don't think they are very intimate; she had never dined at our house before. Aunt Frances only called upon her a little while ago."

"I had never seen her before, and I liked her face; I thought she looked good."

"I dare say she is good," said Edith; "but," she went on a little desperately, "I do not like her."

"Why not?" asked her companion, in surprise.

"She is so tall and grand. She takes away my breath, and makes me feel so small. I am frightened of her. When she is by I always feel such a child, and when she speaks to me I lose my wits, and don't know what to say."

Harry was delighted with this confession; he thought it expressed very charming, girlish timidity; for although he admired Miss Stanford, he did not in the least wish Edith to be like her. He made her a flattering and consolatory answer, which brought them to the door of the old woman they had come to see.

Edith introduced the curate, and the sick woman was glad to see him, and thanked him for coming. Then Edith delivered the contents of her basket, and Harry stood looking on and admiring her graceful and pretty sympathetic manner. After that he talked a little, and then they went away, both promising to come again.

On the homeward way their talk ran on other topics, and they parted the best of friends. From this time they met frequently. Edith seldom took her daily walk to see Mrs. Jones without being joined by the curate at some point. Sometimes she found him at the cottage when she got there, sometimes he arrived just after herself. With every meeting his admiration and attachment grew, until he had begun seriously to consider how to word his offer when the first favourable opportunity for making it should occur.

*(To be continued.)*

